# Factors Affecting Pharmacy Students’ Decision to Study in Pharmacy Colleges in Saudi Arabia: A Cross-Sectional Questionnaire-Based Analysis

**DOI:** 10.3390/healthcare9121651

**Published:** 2021-11-28

**Authors:** Ahmed M. Alshehri, Lara A. Elsawaf, Shaikah F. Alzaid, Yasser S. Almogbel, Mohammed A. Alminggash, Ziyad S. Almalki, Majed A. Algarni

**Affiliations:** 1 Clinical Pharmacy Department, College of Pharmacy, Prince Sattam bin Abdulaziz University, Al Kharj 16273, Saudi Arabia; Lara.ayman.elsawaf@gmail.com (L.A.E.); shaikah.f.alzaid@gmail.com (S.F.A.); Z.almalki@psau.edu.sa (Z.S.A.); 2 Department of Pharmacy Practice, College of Pharmacy, Qassim University, Buraidah 51452, Saudi Arabia; y.almogbel@qu.edu.sa; 3 Ministry of Interior, Medical Services Headquarters, Riyadh 6389, Saudi Arabia; malshehri@moimsd.gov.sa; 4 Clinical Pharmacy Department, College of Pharmacy, Taif University, P.O. Box 11099, Taif 21944, Saudi Arabia; m.alqarni@tu.edu.sa

**Keywords:** pharmacy education, decision to study in pharmacy, selecting pharmacy as a first choice

## Abstract

(1) Background: Many factors may play a role in deciding to opt for pharmacy as a major. However, no previous studies have been conducted in Saudi Arabia to explore these factors. This study aims to identify the potential factors that prompted students to join the pharmacy program. (2) Methods: A cross-sectional questionnaire was distributed among undergraduate pharmacy students in Saudi Arabia, addressing areas such as reasons that encourage them to choose pharmacy as a major, and students’ socio-demographic characteristics. Descriptive statistics were used to describe the study variables, and a simple logistic regression analysis was performed to identify the potential factors. (3) Results: A total of 491 students completed the questionnaire. Around 40% of them had chosen to study pharmacy as their first choice. Only gender, current GPA, and reasons related to the pharmacy field were found to have a statistically significant association with students selecting pharmacy as their first choice. (4) Conclusions: This study shows that pharmacy students have a future-oriented outlook and selected pharmacy as their first choice because it will develop them professionally, financially, and intellectually. Educating high school students about the characteristic of pharmacy would help attract more talented students to the pharmacy carrier.

## 1. Introduction

By increasing the world population, high demand for healthcare professionals, such as pharmacists, is needed to manage people’s health. According to the World Healthcare Organization, in 2010, in developed countries, such as France and the United States (US), there are on average 117 and 93 pharmacists per 100,000, while in developing countries such as the Kingdom of Saudi Arabia there were only 54 pharmacists per 100,000 people [1]. This indicated the need for more pharmacists to accommodate the increase in the population.

The Kingdom of Saudi Arabia has experienced rapid progress in the profession of pharmacy. This is demonstrated by the increasing number of pharmacy colleges and pharmacy graduates. In 1959, King Saud University established Saudi’s first pharmacy college in Riyadh. Four decades later, King Abdulaziz University was established in Jeddah in the western region. A few years later, the course was introduced at King Faisal University, Al-Ahsa in the Eastern region, and King Khalid University, Abha in the Southern region [2,3]. Since then, according to a report published by the Saudi Commission for Health Specialties (SCFHS) in 2018, the number of public and private pharmacy colleges has increased and reached 27, with more than 14,004 pharmacy students enrolled [4]. Thus, the number of graduate pharmacists increased from 150–250 in 2000 to 1157 graduates in 2016 and is expected to increase by 7–10% every year [5,6]. In addition, with the Saudi government’s National Transformation Program (Saudi Vision 2030), the need for pharmacists is increased [7].

Pharmacy degrees provided by these colleges have changed over time. King Saud University’s College of Pharmacy used to offer a Bachelor’s degree in Pharmacy (BPharm) after successful completion of five and a half years; while after its establishment in 2001, King Abdulaziz University’s College of Pharmacy offers a Doctor of Pharmacy (PharmD) degree in six years [8]. Now, almost all colleges offer only PharmD, or are in the transition from BPharm to PharmD, or offer both degrees based on the student’s preferred track. These two programs have slightly different characteristics. The PharmD degree programs reduced some of the basic pharmaceutical sciences such as pharmacognosy and medicinal chemistry and added more clinical studies and rotations [5]. Therefore, these two programs may have prompted students to select pharmacy as their first choice after graduating from high school. However, it is worth mentioning that all colleges of pharmacy in Saudi Arabia allowed students to enter pharmacy directly after graduating from high school, which is different than the admission requirements for pharmacy school in western countries [9,10].

Many studies conducted worldwide have examined the reasons and motivations for students to choose pharmacy as a major. Some studies have shown that students chose pharmacy schools as their first choice, while others have found that students chose it as their second choice. For example, studies in Sudan, Ethiopia, the US, and South Africa have examined students’ selection of pharmacy as the major and shown that the majority of students (79.3%, 67.6%, 53%, and 52.3%, respectively) chose pharmacy as the first choice [11,12,13,14,15].

The reasons why students pick a pharmacy school as their first choice differ from one country to another. For example, in the US and Ethiopia, students were encouraged by a family member, a pharmacist, or a pharmacy student [11,14]. In a western county such as the United Kingdom (UK), students related their decision to study pharmacy to its being a science-based course [16], while in Australia, students related their decision to self-employment and salary [17]. In the South African study, the most important reasons were that they enjoy working with people, want to help poor people and advise them on different types of diseases, and earn decent wages [12]. In the Sudanese study, viewing the career of pharmacy as an excellent option for the future and pharmacists as having a good social image led students to select pharmacy as their first choice [13]. Contrastingly, a study conducted in a Jordanian university showed that almost 61% of all pharmacy students stated that pharmacy school was not their first choice; however, the desire to work in a health-related field led the majority (83.8%) to enroll in a pharmacy college [18]. Receiving encouragement from other people who share the same characteristics was shown to be one of the reasons to study pharmacy [11].

A similar study was conducted in Saudi Arabia at Taif University, and it was found that 62.3% of students (*n* = 398) had applied for the pharmacy program (PharmD) as their second choice after medicine. The same study found that the students were selecting pharmacy as their second choice because they wanted to work in a health-related field (83.4%), had excellent high school grades (73.4%), sought good job opportunities (72.1%), loved to work with patients (70.4%), liked flexible working hours (67.8%), and had received family encouragement (66.6%) [19]. Pharmacists’ basic salary in Saudi Arabia ranged on average from $1979 to $7782 per month and more depending on their practice area, where pharmacists who worked in community pharmacies tend to receive low salaries compared to those who work in hospital pharmacies. However, since Saudi universities offer two pharmacy programs (PharmD and BPharm), examining students’ preferences regarding these two programs is very important.

Students’ reasons for choosing pharmacy as their first choice varied among the studies, thereby identifying different factors that may affect students’ choices. It is important to know why students choose pharmacy school as their first choice after graduating from high school. To our knowledge, no previous studies have been conducted across all regions of Saudi Arabia to explore pharmacy students’ reasons for studying pharmacy and making it their first choice. Therefore, this study aims to identify pharmacy students’ (PharmD and BPharm) reasons for joining the pharmacy program and their career plans.

## 2. Materials and Methods

### 2.1. Study Design, Population, and Samples

A cross-sectional online questionnaire was distributed among a convenient sample of undergraduate pharmacy students in different colleges across Saudi Arabia between January 2021 until July 2021. Since there are 27 colleges of pharmacy located in different universities across Saudi Arabia, and more than 14,000 students are studying pharmacy, two methods were used to distribute the survey and to increase the study participants. First, the questionnaire link was sent to different pharmacy school clubs, and they were asked to distribute it among their undergraduate pharmacy students. Second, the authors sent the questionnaire to pharmacy students at their respective colleges and asked them to share the link with their classmates and other pharmacy students across Saudi Arabia. These two methods were repeated after two months of sending the survey to increase student participation, especially with pharmacy colleges with low response rates.

### 2.2. Study Ethics

Participants were informed at the beginning of the online survey that their participation would be completely voluntary and anonymous, so they could stop the survey at any time and be sure that their information was unidentifiable. In addition, participants were informed that they consented to being included in the study by accepting to complete the survey. The Research Ethics Committee in Health and Science Disciplines (REC-HSD) at Prince Sattam bin Abdulaziz University approved the study.

### 2.3. Survey Instrument

The survey questionnaire was created by modifying various surveys found in the literature [11,18,20,21,22], and it consisted of two parts. The first part focused on the reasons that encouraged the students to choose pharmacy as a major. It included 18 items, and each item needed to be ranked by the student based on its importance. These items were rated using a 5-point Likert scale ranging from “not very important” (1) to “very important” (5). This first part of the survey is divided into three sections. The first section included six items to measure students’ reasons for career advice, such as advice from a high school teacher, a university faculty member, or a pharmacist, and receiving advice while attending events or browsing social media. The second section evaluated personal factors, such as receiving advice from family members or friends, previous pharmacy work experience, desire to work in the healthcare sector or to improve people’s health and well-being, and their grades in high school. The last section included reasons related to the pharmacy field: work flexibility, high salary, ability to run a pharmacy business, high job demand, having a job with important knowledge, and respect.

The second part contained items that asked about students’ socio-demographics variables, such as age, gender, current study year, marital status, university region, current Grade Point Average (GPA), and pharmacy degree program. Additionally, this part asked students whether they had any relatives or friends who worked in health-related fields and whether they had chosen to study Pharmacy as their first choice or not.

The survey was first formed in the English language and then translated to Arabic. To validate the translation, the Arabic version was then translated back to English. Then, the survey was sent to five pharmacy faculty members and 10 pharmacy students to validate the survey. Study item internal consistency reliability was measured through Cronbach’s alpha.

### 2.4. Data Analysis

The study used the G power analysis to determine the required sample size, and the STATA software program to analyze the data produced by the survey. First, various assumptions were utilized to compute the needed sample size: odds ratio, 1.3; power, 0.8; alpha level, 0.05. Based on these assumptions, the required sample size was 473. Next in the Stata analysis, descriptive, simple, and multivariate logistic regression analyses were used to assess the study objectives. Descriptive statistics, including the mean and frequency distribution, were used to describe the study variables. A simple logistic regression analysis was used to determine the association between the independent study variables (age, gender, marital status, pharmacy degree program, first-degree family, current GPA, job advice, personal advice, pharmacy factors) and dependent variable (students’ selecting pharmacy as their first choice after high school). A multivariate logistic regression analysis was used to identify which factors were associated with students selecting pharmacy school as their first choice. A *p*-value of less than 0.05 was considered significant.

## 3. Results

A total of 491 pharmacy students participated in this study. Participants’ average age was 22.26 (±2.31) years, and the majority were female (73.12%) and single (94.50%). The participants’ average GPA was 4.28 (±0.61) on a 5-point scale, and 64.15% were enrolled in a PharmD program while 35.23% were enrolled in a BPharm program. Two-thirds of participants were studying in universities located in the middle region of Saudi Arabia. Most of them (58.65%) had relatives working in health-related fields, and less than half of the participants (41.54%) had selected Pharmacy as their first choice (see Table 1).

### 3.1. Students’ Reasons for Choosing Pharmacy

Table 2 shows students’ reasons for studying in a pharmacy school. Among the three types of reasons, pharmacy students rated reasons related to the pharmacy field as their biggest reason for studying Pharmacy (3.96 ± 0.80), followed by personal factors (3.66 ± 0.78), and lastly, career advice (3.05 ± 0.97). Among reasons related to career advice, participants rated advice from a pharmacist as neutral to important in selecting pharmacy (3.65 ± 1.37), while they rated advice from a schoolteacher as a not important to neutral reason (2.59 ± 1.32). Among reasons related to personal factors, the desire to work in the healthcare sector was perceived as an important to very important reason (4.36 ± 0.99), and indeed, it was the highest-rated reason. Contrastingly, advice from a friend was rated as the least important among reasons related to personal factors (3.04 ± 1.24). Lastly, students rated viewing pharmacy as a leading to a respectable job and a job with important knowledge as an important to very important reason for studying pharmacy (4.30 ± 0.96 and 4.30 ± 1.24, respectively). Subsequently, students rated the ability to run a pharmacy business as neutral to important (3.23 ± 1.28). All the previous domains showed good reliability, ranging from 0.67 to 0.83.

### 3.2. Factors Predicting the Selection of Pharmacy Schools as the First Choice

Simple and multivariate logistic regression analyses were used to determine which factors predicted whether pharmacy students had selected pharmacy school as their first choice after graduation from high school. First, a simple logistic regression analysis examined the association between selecting pharmacy school as the first choice and age, gender, marital status, current GPA, pharmacy degree program, relatives/friends working in the healthcare sector, reasons related to career advice, personal advice, and pharmacy field. Only gender, current GPA, and reasons related to the pharmacy field were found to have a statistically significant association with students selecting pharmacy as their first choice (β = 0.45, *p* < 0.001; β = 2.47, *p* < 0.001; β = 1.04; *p* < 0.035, respectively).

The multivariant logistic regression identified which factors among gender, current GPA, and reasons related to the pharmacy field predicted students’ selection of pharmacy as the first choice after graduating high school. The analysis showed that there was a statistically significant relationship between the variables (χ2 (3, 491) = 0. 619, *p* < 0.001). It showed that there was a significant relationship between students’ selection of pharmacy school as their first choice and their current GPA (*p* < 0.001) and the pharmacy field factors (*p* = 0.017). As a student’s current GPA increased by one unit, the odds of the student selecting pharmacy school as their first choice increased by 2.52 (OR = 2.52, 95% CI = 1.55–4.08, *p* < 0.001). Moreover, as a student’s reasons related to the pharmacy-field factors increased by one unit, the odds of the student selecting pharmacy school as their first choice increased by 2.52 (OR = 1.07, 95% CI = 1.01–1.13, *p* = 0.017) (see Table 3).

## 4. Discussion

To the best of our knowledge, this is the first study involving the investigation of pharmacy students’ reasons behind studying pharmacy as a first choice in Saudi universities. By conducting simple and multivariate logistic regression analyses, we found a positive association between GPA, factors related to the pharmacy field (good job opportunities, a job providing important knowledge, flexible working hours, a good salary, a respectable job, and the ability to run a pharmacy business) and the preference of pharmacy as a first choice.

The study sample’s socio-demographics are slightly different from those of similar studies. The majority of participating students were female (73.12%), which was similar to the proportion of female students in the pharmacy colleges in the United States [23,24,25] and other Arab countries [13,18,26], and different from what was found in the earlier Saudi study [19]. More than half of the participants are currently studying in the central-region universities, most likely because the central region of Saudi Arabia has the highest population density and the highest number of pharmacy colleges [4,27]. Regarding students’ GPA, we found that majority of students had a GPA ranging from 4 to 4.50, or “very good,” which is similar to other studies conducted in Saudi Arabia and Ethiopia [14,19].

Opting for pharmacy after graduation from high school has become one of the common directions for Saudi students in recent years. In our study, 41.5% of the respondents had chosen to study Pharmacy as their first choice, followed by 34.8% and 15.1% who selected it as their second and third choice, respectively. The percentage of students selecting pharmacy school as their first choice was lower than the percentages found in Sudan (79.3%) [13], Ethiopia (67.6%) [14], the United States (53%) [11], and South Africa (52.3%) [12]. On the other hand, although the current study was conducted across different pharmacy schools and among both PharmD and BPharm students, the study’s findings were similar to another study conducted in Saudi Arabia, wherein 37.7% of pharmacy students (PharmD) at Taif University revealed that they had chosen pharmacy school as their first choice [19]. In addition, the present findings were similar to those of other studies conducted in Jordan and South Africa, where only 39% of students had chosen pharmacy as their first choice [15,18]. This highlights the importance of identifying and clarifying the importance and the impact of pharmacists in the community.

Many factors could affect high school graduates’ decisions to study pharmacy in Saudi Arabia. In our study, students rated reasons related to the pharmacy field as the biggest factor that influenced their decision to study Pharmacy. This indicated that students were careful in selecting their careers and were little affected by other factors. Regarding the field of pharmacy-related factors, more than 85% of students perceived having a respectable job with important knowledge as an important to very important reason to choose to study Pharmacy. This indicated students’ interest in keeping up with developments in the pharmacy field and expanding their knowledge even after graduating and getting a job, which was not measured in other studies. However, viewing pharmacy as a respectable job has been reported in the US, since 71% of Americans in 2020 rated pharmacist honesty and ethical standards as “high” or “very high” [28]. 

A study investigating the factors influencing Sudanese pharmacy students to study pharmacy found that 30.5% of participants who chose pharmacy as their first choice did so because it offered a good future; whereas, only 1.9% of them preferred it because it provided a good social image [13]. In our study, among personal factors, around 80% of students rated their desire to work in the healthcare sector and improve people’s health and well-being as “important” to “very important” factors in their decision to study pharmacy. It is very important to note that these two factors could lead students to keep pharmacy as one of the options in the healthcare sector.

Among factors related to career advice, about two-thirds of students rated the advice received from a pharmacist as important to very important, while 47% rated the advice from a schoolteacher as not very important to not important. This indicates that to attract more talented high-school students, pharmacists and pharmacy schools, in general, need to increase their visits to high schools to educate and encourage students to choose pharmacy as a career and to encourage teachers to depict it as an important profession in society. A study showed that students who decided to enter pharmacy school before starting high school were more likely to pursue their plan than students who decided to enter pharmacy while there are in pharmacy school [17]. Contrastingly, another study of pharmacy students at the University of Sierra Leone found that students cited a subject or teacher at school as their primary motivator (66.7%) in opting for pharmacy. In the present study, students considered family and friends as the most significant contributors (61.1%) to their choice. Similar to another study which found that pharmacy students encouraged to study pharmacy school due to a recommendation from their parents and other high-school students who shared similar race [11]. A job with good career opportunities (27.8%), an opportunity for self-employment (27.8%), and working in the healthcare profession with patients (16.7%) were the most valuable career-oriented factors that influenced students’ choice [29]. Having a high salary after graduation was perceived as important by students, similar to the other study [17]. However, it was perceived as less important than perceiving pharmacy as a respectable job with important knowledge.

This study has several limitations. First, the study survey was self-administrated; therefore, students’ responses cannot be validated. Next, since taking the survey was voluntary, some students did not mention their GPA, which they may have perceived as private information. Although the survey was distributed to all universities across the country, students in the central-region universities had the maximum participation, and some pharmacy colleges were not represented because none of their students participated in the survey. Thus, the results cannot be generalized for all universities in the country.

## 5. Conclusions

This study shows that pharmacy students in Saudi Arabia have various reasons to enroll in pharmacy colleges. Students chose pharmacy as their first choice because it would give them good career opportunities and a respectable job with important knowledge, a high salary, and flexible working hours, in addition to the possibility of running a private pharmacy business. The study also shows that pharmacy students have a future-oriented view and desire to obtain a job that will develop them professionally, financially, and intellectually. To improve the future of pharmacy as a career option, faculties pharmacy schools need to visit high schools and provide details regarding the benefits of studying pharmacy and the future of the profession. Future studies may study the effect of pharmacy school visits to high school on students’ understanding of pharmacist-related factors and the impact of these visits on their intention to study pharmacy. Additionally, using a theoretical model like the theory of planned behavior would help identify better the salient factors for studying pharmacy.

## Figures and Tables

**Table 1:** Students’ socio-demographic characteristics (*n* = 491).

Characteristic	*n*	%
Age (years); mean ± *SD*	22.26 (±2.31)	
**Gender**		
Male, *n* (%)	132	26.88
Female, *n* (%)	359	73.12
**Marital status**		
Single, *n* (%)	464	94.50
Married, *n* (%)	23	4.68
Divorced, *n* (%)	0	0
Separated, *n* (%)	4	0.81
Widowed, *n* (%)	0	0
Current Grade Point Average (GPA); mean ± *SD*	4.29 (±0.61)	
**Region of the university**		
Northern region, *n* (%)	39	7.94
Southern region, *n* (%)	55	11.20
Middle region, *n* (%)	326	66.40
Eastern region, *n* (%)	23	4.68
Western region, *n* (%)	61	12.42
**Pharmacy degree program**		
BPharm, *n* (%)	173	35.23
PharmD, *n* (%)	315	64.15
Both (PharmD and BPharm), *n* (%)	3	0.61
**Pharmacy as a choice**		
First choice, *n* (%)	204	41.55
Second choice, *n* (%)	171	34.83
Third choice, *n* (%)	74	15.07
Fourth choice, *n* (%)	7	1.43
Fifth choice, *n* (%)	5	1.02
Not a choice, *n* (%)	36	7.33
**Pharmacy year**		
First year, *n* (%)	6	1.22
Second year, *n* (%)	82	16.70
Third year, *n* (%)	118	24.03
Fourth year, *n* (%)	112	22.81
Fifth year, *n* (%)	106	21.59
Sixth year, *n* (%)	67	13.65
**Relatives/friends working in a health-related field**		
Father, *n* (%)	24	4.89
Mother, *n* (%)	14	2.85
Sister, *n* (%)	109	22.20
Brother, *n* (%)	82	16.70
Husband, *n* (%)	1	0.20
Relatives, *n* (%)	288	58.66

**Table 2:** Students’ reasons for choosing pharmacy as a major (*n* = 491).

Items	Mean (±*SD*)	Frequency *n* (%)				
		Not Very Important	Not Important	Neutral	Important	Very Important
**Career Advice**						
Advice from a schoolteacher	2.59 (±1.32)	149 (30.35)	83 (16.90)	113 (23.01)	111 (22.61)	35 (7.13)
Advice from a university faculty member	3.17 (±1.44)	105 (21.38)	56 (11.41)	83 (16.9)	143 (29.12)	104 (21.18)
Advice from a pharmacist	3.65 (±1.37)	68 (13.85)	34 (6.92)	62 (12.63)	161 (32.79)	166 (33.81)
Advice received while attending a recruitment event	3.07 (±1.32)	96 (19.55)	53 (10.79)	132 (26.88)	139 (28.31)	71 (14.46)
Self-directed career advice from internet searches	2.96 (±1.23)	88 (17.92)	74 (15.07)	146 (29.74)	138 (28.11)	45 (9.16)
Advice from social media	2.85 (±1.25)	102 (20.77)	71 (14.46)	165 (33.60)	105 (21.38)	48 (9.78)
Domain Total	3.05 (±0.97)			Cronbach’s alpha = 0.83		
**Personal Factors**						
Advice from a family member	3.42 (±1.35)	74 (15.07)	46 (9.37)	87 (17.72)	168 (34.22)	116 (23.63)
Advice from a friend	3.04 (±1.24)	88 (17.92)	59 (12.02)	138 (28.11)	159 (3238)	47 (9.57)
Previous pharmacy work experience	3.25 (±1.53)	115 (23.42)	47 (9.57)	63 (12.83)	132 (26.88)	134 (27.29)
Desire to improve people’s health and well-being	4.11 (±1.18)	39 (7.94)	10 (2.04)	52 (10.59)	148 (30.14)	242 (49.29)
Desire to work in the healthcare sector	4.36 (±0.99)	21 (4.28)	8 (1.63)	32 (6.52)	144 (29.33)	286 (58.25)
High school grades	3.79 (±1.25)	45 (9.16)	28 (5.70)	88 (17.92)	152 (30.96)	178 (36.25)
Domain Total	3.66 (±0.78)			Cronbach’s alpha = 0.67		
**Pharmacy Factors**						
Flexible work hours	3.68 (±1.17)	40 (8.15)	34 (6.92)	97 (19.76)	194 (39.51)	126 (25.66)
High salary after graduation	4.07 (±1.03)	20 (4.07)	23 (4.68)	55 (11.20)	200 (40.73)	193 (39.31)
Ability to run pharmacy business	3.23 (±1.28)	59 (12.02)	84 (17.11)	126 (25.66)	128 (26.07)	94 (17.14)
Good job opportunities	4.16 (±1.45)	25 (5.09)	11 (2.24)	52 (10.59)	177 (36.97)	226 (46.03)
A job with important knowledge	4.30 (±1.24)	23 (4.68)	9 (1.83)	41 (8.35)	145 (29.53)	273 (55.60)
Respectable job	4.30 (±0.96)	19 (3.87)	8 (1.63)	38 (7.74)	168 (34.22)	258 (52.55)
Domain Total	3.96 (±0.80)			Cronbach’s alpha = 0.83		

**Table 3:** Simple and multivariate logistic regression analysis of factors predicting pharmacy students’ selecting pharmacy school as their first choice (*n* = 491).

Variable	*p*-Value	Odds Ratio	95% Confidence Interval	
			Lower	Upper
**Simple Logistic Regression**				
Age	0.921	1.14	0.90	1.12
Gender	<0.000 *	0.45	0.30	0.70
Marital status	0.293	1.57	0.68	3.63
Pharmacy degree program	0.352	0.84	0.58	1.22
First-degree family	0.363	1.19	0.82	1.72
Current GPA^2^	<0.000 *	2.47	1.54	3.96
Job advice	0.760	1.48	0.97	1.04
Personal advice	0.583	1.01	0.97	1.05
Pharmacy factors	0.035 *	1.04	1.311	1.09
**Multivariate Logistic Regression**				
Gender	0.354	0.76	0.43	1.35
Current GPA^2^	<0.001 *	2.52	1.55	4.08
Pharmacy field factors	0.017 *	1.07	1.01	1.13

Note: *p*-value < 0.05 indicated with asterisk.

## Data Availability

The data that support the findings of this study are available from the corresponding author.
